# Thermoelectric Properties of Ca_3_Co_2−*x*_Mn*_x_*O_6_ (*x* = 0.05, 0.2, 0.5, 0.75, and 1)

**DOI:** 10.3390/ma12030497

**Published:** 2019-02-06

**Authors:** Nikola Kanas, Sathya Prakash Singh, Magnus Rotan, Temesgen Debelo Desissa, Tor Grande, Kjell Wiik, Truls Norby, Mari-Ann Einarsrud

**Affiliations:** 1 Department of Materials Science and Engineering, NTNU Norwegian University of Science and Technology, NO-7491 Trondheim, Norway; nikola.kanas@ntnu.no (N.K.); sathya.p.singh@ntnu.no (S.P.S.); magnus.rotan@ntnu.no (M.R.); tor.grande@ntnu.no (T.G.); kjell.wiik@ntnu.no (K.W.); 2 Department of Chemistry, University of Oslo, FERMiO, Gaustadalléen 21, NO-0349 Oslo, Norway; t.d.desissa@smn.uio.no (T.D.D.); truls.norby@kjemi.uio.no (T.N.)

**Keywords:** Ca_3_Co_2−*x*_Mn*_x_*O_6_, thermoelectric properties, thermal conductivity, electrical conductivity, Seebeck coefficient

## Abstract

High-temperature instability of the Ca_3_Co_4−*y*_O_9+*δ*_ and CaMnO_3−*δ*_ direct p-n junction causing the formation of Ca_3_Co_2−*x*_Mn*_x_*O_6_ has motivated the investigation of the thermoelectric performance of this intermediate phase. Here, the thermoelectric properties comprising Seebeck coefficient, electrical conductivity, and thermal conductivity of Ca_3_Co_2−*x*_Mn*_x_*O_6_ with *x* = 0.05, 0.2, 0.5, 0.75, and 1 are reported. Powders of the materials were synthesized by the solid-state method, followed by conventional sintering. The material Ca_3_CoMnO_6_ (*x* = 1) demonstrated a large positive Seebeck coefficient of 668 μV/K at 900 °C, but very low electrical conductivity. Materials with compositions with *x* < 1 had lower Seebeck coefficients and higher electrical conductivity, consistent with small polaron hopping with an activation energy for mobility of 44 ± 6 kJ/mol and where both the concentration and mobility of hole charge carriers were proportional to 1−*x*. The conductivity reached about 11 S·cm^−1^ at 900 °C for *x* = 0.05. The material Ca_3_Co_1.8_Mn_0.2_O_6_ (*x* = 0.2) yielded a maximum *zT* of 0.021 at 900 °C. While this value in itself is not high, the thermodynamic stability and self-assembly of Ca_3_Co_2−*x*_Mn*_x_*O_6_ layers between Ca_3_Co_4−*y*_O_9+*δ*_ and CaMnO_3−*δ*_ open for new geometries and designs of oxide-based thermoelectric generators.

## 1. Introduction

Ceramics based on cobalt oxides are attractive for many applications [1] because the wide variation in crystal structure, oxygen non-stoichiometry, and valence of Co give rise to a wide range of useful properties [2]. Ca_3_Co_4_O_9+*δ*_ (CCO) is among the best p-type thermoelectric (TE) materials and also a potential cathode material for solid oxide fuel cells [3]. Ca_3_Co_2_O_6_ (C326) has a lower TE performance compared to CCO, but is stable up to 1026 °C in ambient air, which is ~100 °C higher than the stability limit for CCO [4,5]. Due to the higher stability, C326 has been studied in order to understand and improve its ferroelectric, magnetic, and thermoelectric properties [6,7,8,9,10]. C326 has the A_3_BB’O_6_ structure (A = alkaline earth and B = transition metal or alkaline earth) [8] consisting of chains of alternating face-sharing CoO_6_ octahedra (Co1° positions) and trigonal biprisms (Co2^t^ positions) with Ca ions between the chains [11]. The special arrangement of Co–O–Co makes C326 a narrow-band semiconductor with strong anisotropy where the charge transport is more dominant in the *a-b* plane than along the *c* direction [9]. Pure C326 has a positive Seebeck coefficient of about 170 μV/K reported by Mikami et al. [12] and about 180 μV/K reported by Iwasaki et al. [13] at 700 °C. C326 has electrical conductivity of about 5 S·cm^−1^ at 500 °C [14], and at ambient temperature a thermal conductivity of about 1.7 W·m^−1^·K^−1^ [8] is reported. Improvements of the properties have been reported by substitution on A(Ca)- and B(Co)-sites and a significant enhancement of the electrical conductivity was observed when 50 at.% of Co was substituted with Ir [8]. A 10 at.% Cu substitution on the Co-site gave a significant improvement in the power factor [13]. Hervoches et al. [11] investigated the effect of Mn substitution on the Co-site in terms of structural and magnetic properties, and found that the crystal structure belongs to the R3c space group with unit cell dimensions of 9.084 Å < *a* < 9.134 Å and 10.448 Å < *c* < 10.583 Å for Ca_3_CoMnO_6_ [11]. They also confirmed a solid solution in the entire composition interval (0 < *x* < 1) [11] by using sol-gel synthesis, in contrast to results obtained by solid-state synthesis by Bazuev et al. [15], where an inhomogeneity region at about *x* = 0.5 was observed [15,16]. For Ca_3_CoMnO_6_, there is complete filling of Co1^o^ by Mn [11]. Hervoches et al. showed presence of cation ordering for the composition with *x* = 1, while for those with *x* < 1 cation disordering [11]. They also state that the Mn has the oxidation state 4+, hence converting the Co2^t^ occupancies to Co^2+^ [11]. Mikami et al. demonstrated that 10 at.% Mn substitution on the Co-site caused an increase in electrical conductivity in the low-temperature region, but a decrease in the power factor due to a significant reduction of the Seebeck coefficient [12]. High stability of Ca_3_Co_2−*x*_Mn*_x_*O_6_ for 0 ≤ *x* ≤ 0.25 has been reported by Golovkin and Bazuev [4]. 

In our previous work on the CCO-CaMnO_3_-based oxide p-n junction, Ca_3_Co_2−*x*_Mn*_x_*O_6_ with different contents of Mn were formed at the CCO-CaMnO_3_ interface after heat treatment at 900 °C [17]. Furthermore, the CCO-CaMnO_3_ oxide p-n junction was also utilized in the recently developed all-oxide TE module, where Ca_3_Co_2−*x*_Mn*_x_*O_6_ was formed as a reaction product at the CCO-CaMnO_3_ p-n junction affecting the electrical conductivity across the junction, as well as boosting the generated voltage across the hot side of the TE module [17].

To the best of our knowledge there are no reports describing the TE properties of Ca_3_Co_2-*x*_Mn*_x_*O_6_ in the whole composition range (0 < *x* ≤ 1) within the temperature range of 200–900 °C, and we here report a detailed study of the electrical conductivity, thermal conductivity and Seebeck coefficient of Ca_3_Co_2−*x*_Mn*_x_*O_6_ (*x* = 0.05, 0.2, 0.5, 0.75, and 1) materials. The TE properties of this phase may help to understand and optimize performance of all-oxide thermoelectric devices based on CCO and CaMnO_3_ [17]. 

## 2. Materials and Methods 

### 2.1. Materials Synthesis

Ca_3_Co_2−*x*_Mn*_x_*O_6_ powders (*x* = 0.05, 0.2, 0.5, 0.75, and 1) were synthesized by the solid-state method using submicron CaCO_3_, Mn_2_O_3_ and Co_3_O_4_ precursors from Inframat Advanced Materials, (Manchester, CT, USA, >99% purity). The precursors were first dried at 120 °C for 5 h to remove adsorbed moisture and perform accurate weighing for five different compositions, and then each batch was ball-milled in isopropanol for 8 h using zirconia balls in order to obtain homogeneous mixtures. After drying in a rotavapor (R-210, Büchi, Bern, Switzerland), the powder mixtures were uniaxially pressed into pellets (one for each batch) at 5 MPa and heated at 1000 °C for 10 h in ambient air. After grinding and reheating the pellets using the same procedure, the final powders were prepared by grinding the reheated pellets in a mortar followed by sieving. The final powders were then pressed into bars (15 × 5 × 2 mm^3^) and pellets (diameter 12.7 mm, thickness 2.2 mm) by cold isostatic pressing at 200 MPa, and conventionally sintered in ambient air at 1010 °C for 30 h, using heating and cooling rates of 200 °C/h.

### 2.2. Characterization

X-ray diffraction (XRD, Bruker, Billerica, MA, USA) of sintered and ground pellets was performed to validate the phase purity of the materials. The data were recorded on a Bruker D8 Advance equipped with a Lynxeye XE detector using a step size of 0.013° and counting 16.9 s/step. Unit cell parameters of Ca_3_Co_2−*x*_Mn*_x_*O_6_ were refined using Topas 5 software (Bruker, MA, USA) by Pawley fitting. Morphology and particle size were determined by SEM (S-3400N, Hitachi, Tokyo, Japan). The thermal expansion coefficients (TECs) during heating and cooling were recorded in the range 100–800 °C in ambient air using a dilatometer (DIL 402, Netzsch, Cologne, Germany). The densities of the ceramic samples were measured by the Archimedes method in isopropanol. Thermal conductivity was acquired by the laser flash method (LFA 457 MicroFlash, Netzsch, Cologne, Germany) on pellet-shaped samples [18], while Seebeck coefficient and electrical conductivity were measured on bar-shaped samples using a four-point method [19,20]. All TE data were collected in the temperature range 200–900 °C with steps of 100 °C. 

## 3. Results and Discussion

### 3.1. Materials Characteristics

**Figure 1:**
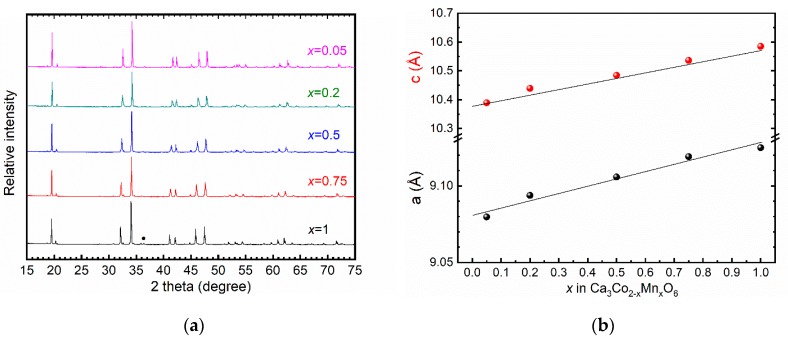
(**a**) XRD patterns of Ca_3_Co_2−*x*_Mn*_x_*O_6_ (*x* = 0.05, 0.2, 0.5, 0.75, and 1) with marked Mn_2_O_3_ peak (•); and (**b**) cell parameters as a function of *x* in Ca_3_Co_2−*x*_Mn*_x_*O_6_. Lines represent corresponding data reported by Hervoches et al. for materials prepared by the sol-gel method [11].

XRD patterns of sintered samples of Ca_3_Co_2–*x*_Mn*_x_*O_6_ with *x* = 0.05, 0.2, 0.5, 0.75 and 1 are shown in Figure 1a.

Diffraction lines for the target phase Ca_3_Co_2−*x*_Mn*_x_*O_6_ were indexed according to the reference patterns for the compositions Ca_3_CoMnO_6_ [6], Ca_3_Co_1.25_Mn_0.75_O_6_ [11], Ca_3_Co_1.5_Mn_0.5_O_6_ [11], Ca_3_Co_1.8_Mn_0.2_O_6_ [12] and Ca_3_Co_1.95_Mn_0.05_O_6_ [14]. Phase-pure materials were obtained showing that by careful synthesis the formation of secondary phases can be avoided, as opposed to the previously published difficulties in obtaining phase pure materials in this system using solid-state synthesis [6,15]. An additional low-intensity diffraction line in the Co_3_CoMnO_6_ (*x* = 1) pattern was observed at 2θ ≈ 36.5°, which corresponds to Mn_2_O_3_. The variation in cell parameters of Ca_3_Co_2−*x*_Mn*_x_*O_6_ with increasing *x* is in good agreement with the data previously reported by Hervoches et al. [11] according to Figure 1b. The lattice parameters are summarized in Table 1. 

**Figure 2:**
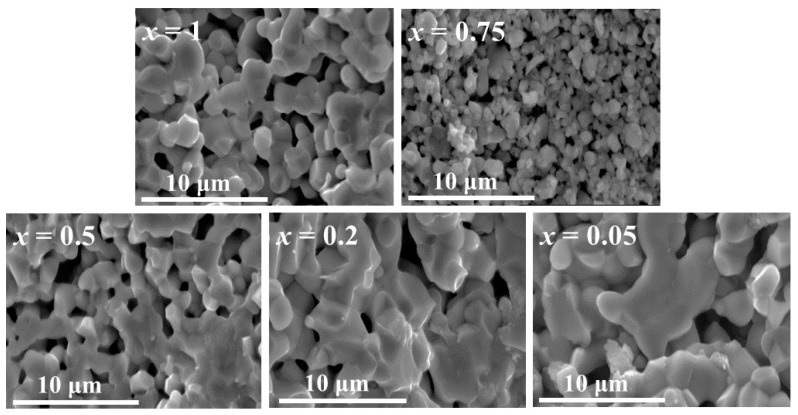
SEM micrographs of Ca_3_Co_2−*x*_Mn*_x_*O_6_ fracture surfaces, *x* = 1, 0.75, 0.5, 0.2, and 0.05.

Microstructures of the ceramic materials with different *x* are presented in Figure 2. An increase in grain coarsening with decreasing Mn-content (*x*) is observed, where the smallest *x* resulted in microstructure with the largest grains. Ca_3_Co_2_O_6_ naturally forms non-spherical (polyhedral) grains [14] which predominantly intend to grow more in a-b than in c-direction due to its crystal structure [6]. Hence, the increasing grain size of Ca_3_Co_2−*x*_Mn*_x_*O_6_ when decreasing *x* towards 0 (Ca_3_Co_2_O_6_) might result in modifications in grain size and morphology. Furthermore, the microstructures show more inter-connected grains with decreasing *x*. Moreover, the microstructure of the material with *x* = 1 contains larger and more inter-connected grains than the material with *x* = 0.75. 

The bulk densities of the sintered materials were in the range 3.15–3.43 g/cm^3^ giving 73 to 76% relative density, using crystallographic densities calculated from Hervoches et al. [11], as summarized in Table 1. TECs of the materials recorded in the temperature intervals 100–400 °C and 400–800 °C during heating and cooling were in the range 14.4–16.2 × 10^−6^ K^−1^ and 18–20 × 10^−6^ K^−1^, respectively (Table 1).

### 3.2. Thermoelectric Properties

**Figure 3:**
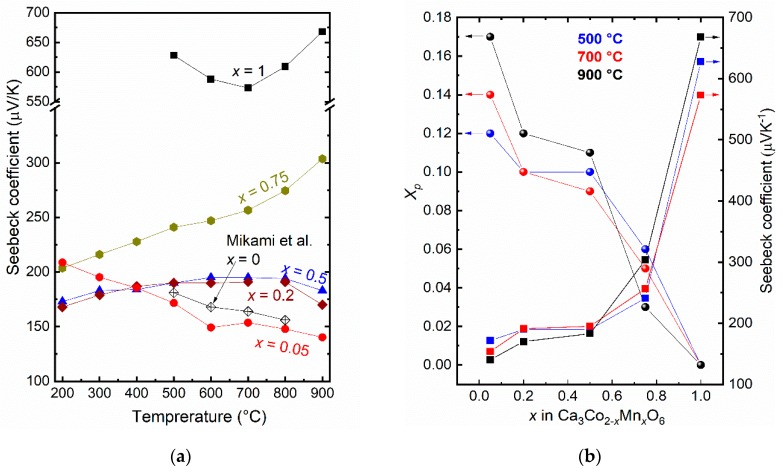
Seebeck coefficient of Ca_3_Co_2−*x*_Mn*_x_*O_6_ as a function of (**a**) temperature and (**b**) *x* at selected temperatures. Data for Ca_3_Co_2−*x*_Mn*_x_*O_6_ (*x* = 0), from Mikami et al. [12], are included for comparison. Figure 3b includes a plot of *X_p_* (left hand axis) based on Equation (2), which is calculated from Seebeck coefficients at 500, 700, and 900 °C.

The Seebeck coefficients of Ca_3_Co_2−*x*_Mn*_x_*O_6_ are presented as a function of temperature and *x* in Figure 3a,b, respectively. They are all positive, showing predominantly p-type conductivity for all conditions and compositions. The Seebeck coefficient increases with increasing *x* while it is to a first approximation not very affected by temperature, at least for the *x* ≤ 0.5 samples. This suggests that charge carriers are not generated by a thermally activated process, but present in the form of partially filled states connected to cobalt, or by a reaction such as

(1) $$2Co^{3+}=Co^{2+}+Co^{4+}$$

(disproportionation) being driven to complete disorder by having a negligible enthalpy. This will give a fraction of Co^4+^, representing electron holes, not higher than 1/3 (Co^4+^ over all Co^3+^+Co^4+^), and more reasonably somewhat lower by e.g., only one of the two Co sites disproportionated or degeneracy by spin or coupling to Co–O bonds.

We shall see that the electrical conductivity indicates a hopping mobility of charge carriers, and we may therefore apply Heikes’ formula [21] to get a first approximation of the fraction of Co from Co1° site contributing to the p-type conductivity based on the Seebeck coefficients:

(2) $$S=\frac{k_{B}}{e}\ln(\frac{1-X_{p}}{X_{p}})$$

Here, the Seebeck coefficient *S* is a function of Boltzmann’s constant *k_B_*, the (elementary) charge of the charge carrier (holes) *e*, and the fraction of Co from Co1° site contributing to the p-type conductivity *X_p_*. Figure 3b (left hand axis) shows the apparent fraction of holes *X_p_* based on data at 500, 700, and 900 °C, starting with reasonable values in the range 1/8–1/6 for the Co-rich compositions (*x* = 0.05) and decreasing quite linearly with *x* and approaching zero for *x* = 1. From this we may state quite unambiguously that the charge carrier (hole) concentration is directly proportional to 1 − *x*, i.e., the concentration of Co on the Co1° site. Ideally, the *x* = 1 composition must then generate its charge carriers by a different, e.g., thermally activated mechanism.

**Figure 4:**
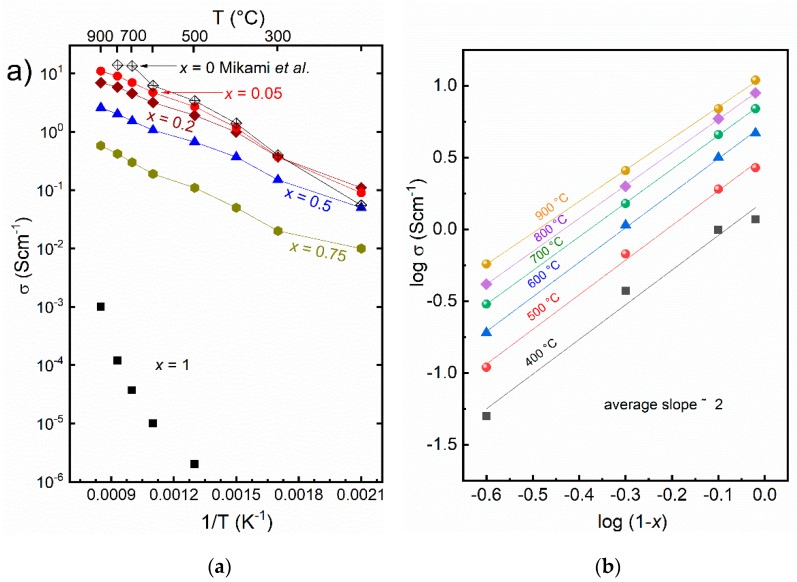
(**a**) Arrhenius plot of electrical conductivity of Ca_3_Co_2-*x*_Mn*_x_*O_6_ versus 1/T. The data for Ca_3_Co_2−*x*_Mn*_x_*O_6_ (*x* = 0) are from Mikami et al. [12]. (**b**) Double-logarithmic plot of isothermal conductivities from 400 to 900 °C versus (1 − *x*) suggesting a slope of 2 at the higher temperatures.

Figure 4 shows an Arrhenius plot of the electrical conductivity of Ca_3_Co_2−*x*_Mn*_x_*O_6_ which increases in a simple activated manner with increasing temperature for all compositions *x* < 1. In combination with the high, un-activated concentrations of charge carriers deduced from the Seebeck coefficients, it is reasonable to interpret the activated conductivity in terms of a small polaron hopping model [22]:

(3) $$\sigma =\frac{\sigma_{0}}{T}\exp(-\frac{E_{a}}{RT})$$

Accordingly, fitting ln(*σT*) vs. 1/*T* (not shown) yields pre-exponentials *σ*_0_ and activation energies *E_a_*, and these are included in Table 1. The activation energies fall in the range 44 ± 6 kJ/mol, fairly independent of composition for *x* < 1, and we suggest that this may be attributed to the barrier of electron hole hopping between Co sites.

The pre-exponential *σ*_0_ increases with decreasing *x*, as expected from the behavior of the concentration of charge carriers deduced from the Seebeck coefficient. However, plots of the pre-exponential, or more sensibly, isothermal conductivities, versus 1 − *x* do not yield simple linear relationships. Figure 4b is a double-logarithmic plot suggesting a slope of 2, i.e., the conductivity is proportional to (1 − *x*)^2^. Since we have already concluded that the concentration of charge carriers is proportional to 1 − *x*, the square dependency suggests further that also the charge mobility is proportional to 1 − *x*. This is quite reasonable; the electron holes can jump on Co1° but not Mn1° occupants. Alternatively, one may take the broader approach that the Mn^4+^1°–Co^2+^2^t^ combination—unlike the Co^3+^1°–Co^3+^2^t^ combination—cannot host hole jumps. The *x* = 1 composition is left with a low and in the ideal case activated concentration of charge carriers—giving the high Seebeck coefficient of around 600 μV/K—while the mobility becomes low and the resulting conductivity very low. 

**Figure 5:**
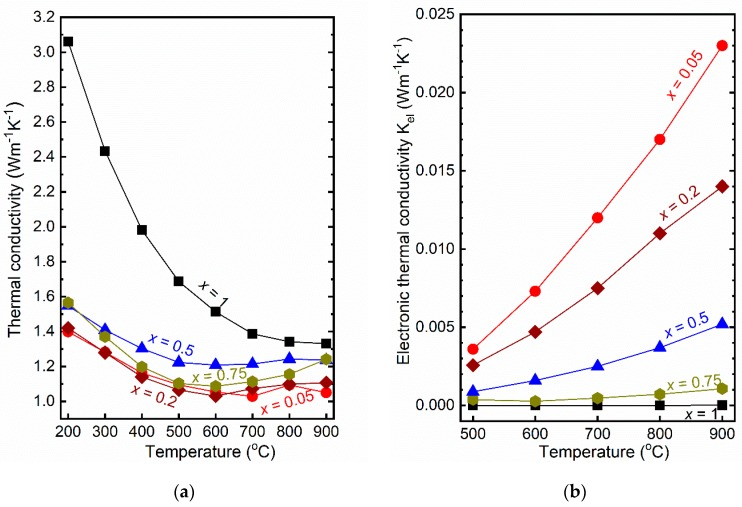
(**a**) Thermal conductivity of Ca_3_Co_2−*x*_Mn*_x_*O_6_, *x* = 0.05, 0.2, 0.5, 0.75, and 1 as a function of temperature; and (**b**) calculated electronic thermal conductivity as a function of temperature.

The thermal conductivity of Ca_3_Co_2−*x*_Mn*_x_*O_6_ given in Figure 5a decreases with increasing temperature for all materials. The material with *x* = 1 reveals the highest thermal conductivity of about 3 W·m^−1^·K^−1^ at 200 °C, while 1 W·m^−1^·K^−1^ at 600 °C represents the lowest value, obtained for *x* = 0.2. In spite of similar relative porosities, the composition with *x* = 1 demonstrated significantly higher thermal conductivity than the other materials. This may be attributed to the ordered cation arrangement at Co1° for Ca_3_CoMnO_6_ (*x* = 1) [11] as opposed to the Co-Mn disorder in the solid solutions of 0 < *x* < 1.

In general, thermal conductivity is the sum of electronic, *κ_el_*, and phonon, *κ_p_*, (lattice) thermal conductivities, where the electronic part can be calculated by Equation (4)

(4) $$\kappa_{el}=\sigma TL$$

where *σ* is the total electrical conductivity (S·cm^−1^), *T* absolute temperature (K) and *L* is the Lorenz number (W·Ω·K^−2^) [23] which can be calculated by Equation (5)

(5) $$L=1.5+\exp\left(-\frac{\left|S\right|}{116}\right)$$

where *S* represents the Seebeck coefficient [24]. The calculated electronic part of the thermal conductivity of Ca_3_Co_2−*x*_Mn*_x_*O_6_ as a function of temperature presented in Figure 5b is increasing with decreasing *x* and temperature in accordance with the relative variation in electronic conductivity. The highest electronic thermal conductivity is observed at 900 °C for *x* = 0.05, which represents only 2% of the total thermal conductivity, the rest being dominated by the phonon contribution. 

**Figure 6:**
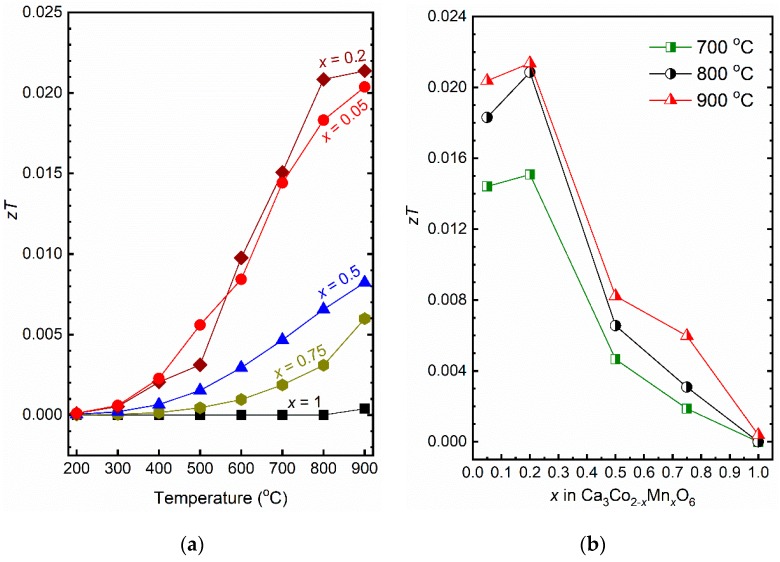
(**a**) Figure of merit *zT* of Ca_3_Co_2−*x*_Mn*_x_*O_6_, *x* = 0.05, 0.2, 0.5, 0.75, and 1 as a function of temperature and (**b**) as a function of *x* in Ca_3_Co_2−*x*_Mn*_x_*O_6_ at 700, 800 and 900 °C.

The figures of merit (*zT*), calculated from Equation (6)

(6) $$zT=\frac{\sigma S^{2}T}{\kappa }$$

are presented in Figure 6a. The *zT* values increased with increasing temperature and the highest value of 0.021 was obtained for *x* = 0.2 at 900 °C, due to a combination of relatively low thermal conductivity and high electrical conductivity as well as a moderate Seebeck coefficient.

The *zT* values as a function of *x* presented in Figure 6b decrease with increasing *x* in the temperature range of 700–900 °C. The material with *x* = 0.2 demonstrated a promising *zT* value which probably can be further improved by using alternative synthesis methods (such as sol-gel or spray pyrolysis) followed by e.g. spark plasma sintering to obtain smaller grains and a further reduction in thermal conductivity due to enhanced phonon scattering at grain boundaries. The porosity of the materials will also influence the thermal and electrical conductivity. Pore size and distribution as well as type of pores could be tailored is such a way to reduce thermal conductivity more than the power factor, to enhance *zT* [25]. Designing a continuous mesoporous nanocrystalline framework seems to be a promising solution [25].

## 4. Conclusions

Phase pure Ca_3_Co_2−*x*_Mn*_x_*O_6_ materials with *x* = 0.05, 0.2, 0.5, 0.75, and 1 were prepared by a solid-state synthesis method. Cold isostatic pressing followed by conventional sintering resulted in relative bulk densities in the range of 73–76% of theoretical. The Seebeck coefficient shows that the materials are p-type conductors, seemingly by holes from fully disordered Co oxidation states. This concentration hence decreases with *x* and the Seebeck coefficient correspondingly increases and reaches values well above 600 μV/K^−1^ for Ca_3_CoMnO_6_ at high temperatures. For *x* < 1, the electrical conductivity is consistent with small polaron hopping with an activation energy of mobility of 44 ± 6 kJ/mol. The conductivity increases with decreasing *x*, reaching above 10 S·cm^−1^ at 900 °C for *x* ≤ 0.05. It appears that the conductivity is proportional to (1 − *x*)^2^, which suggests that both the concentration and mobility of charge carriers are proportional to the Co content on the Co1° site (1 − *x*), where the electron holes can jump on Co1° sites but not Mn1° sites. Thermal conductivity is dominated by phonon transport, with only minor electronic contribution. The figure-of-merit increased with decreasing Mn-content (*x*) till a maximum at *x* = 0.2 of *zT =* 0.021. While this is in itself modest, the thermodynamic stability and in situ formation of Ca_3_Co_2−x_Mn_x_O_6_ between Ca_3_Co_4−*y*_O_9+*δ*_ and CaMnO_3−*δ*_ open for new geometries and designs of oxide-based thermoelectric generators.

## Figures and Tables

**Table 1:** Cell parameters, cell volume, bulk density, thermal expansion coefficient, activation energy and pre-exponential factor of electric conductivity, and figure of merit of sintered Ca_3_Co_2−*x*_Mn*_x_*O_6_ materials with different Mn content (*x*).

Ca_3_Co_2−*x*_Mn*_x_*O_6_	*x* = 1	*x* = 0.75	*x* = 0.5	*x* = 0.2	*x* = 0.05	*x* = 0
a parameter (Å)	9.1312	9.1192	9.1058	9.0938	9.0797	9.0850 *
c parameter (Å)	10.5793	10.5362	10.4845	10.4391	10.3893	10.3888 *
Cell volume (Å^3^)	763.9	758.8	752.9	747.6	741.8	737.6 *
Bulk density (g/cm^3^)	3.15	3.21	3.26	3.42	3.43	-
Crystallographic density (g/cm^3^)	4.31 *	4.34 *	4.40 *	4.48 **	4.51 **	4.52 *
Relative density (%)	73	73	74	76	76	-
TEC 400–800 °C, (10^−6^ K^−1^)	19.8	18	19.2	20	20	-
TEC 800–400 °C, (10^−6^ K^−1^)	19.7	18.2	19.2	19.7	20.7	-
TEC 100–400 °C, (10^−6^ K^−1^)	14.4	14.8	15.6	16.2	15.9	-
TEC 400–100 °C, (10^−6^ K^−1^)	15.0	14.5	15.6	15.9	15.8	-
Activation energy *E*_a_ (kJ/mol)	-	45.9	38.4	39.0	42.9	50.1 ^#^
*σ*_0_ / 1000 (S·K/cm)	-	33	73	220	490	1800 ^#^
Figure of merit *zT* at 900 °C	0.0004	0.006	0.008	0.021	0.020	-

* Calculated from data by Hervoches et al. [11] and ^#^ by Mikami et al. [12]. ** Interpolated data.
